# Double emulsions for iron encapsulation: is a high concentration of lipophilic emulsifier ideal for physical and chemical stability?

**DOI:** 10.1002/jsfa.9691

**Published:** 2019-04-15

**Authors:** Patrícia Duque‐Estrada, Eefje School, Atze Jan van der Goot, Claire C Berton‐Carabin

**Affiliations:** ^1^ Food Process Engineering Wageningen University & Research Wageningen The Netherlands

**Keywords:** double emulsions, polyglycerol polyricinoleate, lipid oxidation, ferrous sulphate, encapsulation

## Abstract

**BACKGROUND:**

Worldwide iron deficiency in diets has led to a growing interest in the development of food‐compatible encapsulation systems for soluble iron, which are able to prevent iron's undesirable off‐taste and pro‐oxidant activity. Here, we explore the use of double emulsions for this purpose, and in particular, how the lipophilic emulsifier (polyglycerol polyricinoleate, PGPR) concentration influences the physicochemical stability of water‐in‐oil‐in‐water (W_1_/O/W_2_) double emulsions containing ferrous sulphate in the inner water droplets. Double emulsions were prepared with sunflower oil containing 10 to 70 g kg^−1^ PGPR in the oil phase, and were monitored for droplet size distribution, morphology, encapsulation efficiency (EE) and oxidative stability over time.

**RESULTS:**

Fresh double emulsions showed an initial EE higher than 88%, but EE decreased upon storage, which occurred particularly fast and to a high extent in the emulsions prepared with low PGPR concentrations. All double emulsions underwent lipid oxidation, in particular those with the highest PGPR concentration, which could be due to the small inner droplet size and thus promoted contact between oil and the internal water phase.

**CONCLUSION:**

These results show that a too high PGPR concentration is not needed, and sometimes even adverse, when developing double emulsions as iron encapsulation systems. © 2019 The Authors. *Journal of The Science of Food and Agriculture published* by John Wiley & Sons Ltd on behalf of Society of Chemical Industry.

## INTRODUCTION

Iron deficiency is one of the most common global nutritional deficiencies and an important factor responsible for anaemia.^1^ To prevent iron deficiency, iron fortification in diets is one of the approaches that should be considered. Yet, iron fortification of food products is still a challenge, because of iron's pro‐oxidant activity. Among the chemical forms of iron available for food fortification, water‐soluble forms have the highest bioavailability. However, soluble iron can readily catalyse oxidation reactions, leading to detrimental sensory and nutritional changes.^2^ Furthermore, soluble iron gives an undesirable metallic taste.^2^ Therefore, encapsulation has been described as a strategy to improve iron stability in food products and to mask its metallic taste.

Among the plethora of food‐compatible encapsulation systems, water‐in‐oil‐in‐water (W_1_/O/W_2_) double emulsions are suitable to encapsulate water‐soluble compounds.^3^ Double emulsions combine a high encapsulation efficiency potential with the ability to mask undesirable tastes in a simple, low‐cost method.^4^ Only a few studies have focused on encapsulating iron within water‐in‐oil (W/O) emulsions^5^, ^6^, ^7^ and within double emulsions.^7^, ^8^, ^9^, ^10^, ^11^ Although these studies have shown that double emulsions are suitable to encapsulate iron with a high and stable encapsulation efficiency (EE), and in amounts that are relevant to food fortification, they have also pointed out that the presence of iron in water droplets dispersed in oil largely favours lipid oxidation, which was to be expected due to the strong pro‐oxidant activity of iron.^12^, ^13^, ^14^

Although attempts have been made at mitigating the pro‐oxidant effect of encapsulated iron in such W/O or double emulsions, for example by increasing the solid fat content of the lipid phase,^5^ the effect of other potentially determining factors, such as the water droplet size, remains unexplored. The contact area between oil and iron‐loaded inner droplets in double emulsions is probably a factor that can affect lipid oxidation, and directly depends on the inner droplet size. The latter can be controlled by processing conditions, and by the type and concentration of lipophilic emulsifier.^15^ The most frequently used lipophilic emulsifier to stabilize water droplets in oil, for food applications, is polyglycerol polyricinoleate (PGPR), which is known to facilitate the production of small water droplets with a narrow size distribution.^16^

Although how PGPR concentration may affect the chemical stability of double emulsions (i.e. lipid oxidation) has not been investigated yet, it is well established that the PGPR concentration affects the EE value in such systems. For instance, a double emulsion is considered reasonably stable if EE is ∼95% (or above) and decrease to 70–80%, maximally, after a few weeks of storage.^17^ Su *et al.* 18 found that in fresh double emulsions prepared with 80 g L^−1^ PGPR in the oil phase, the EE was almost 100% while with 5 g L^−1^ PGPR, almost 48% of the Poly R‐478 dye remained encapsulated. Increased PGPR concentration can create a stable interfacial layer at the water–oil interface and increase the oil phase viscosity, which reduces coalescence of water droplets in the W/O emulsion. These aspects contributed to reducing the release of Poly R‐478 dye at high PGPR concentration. It does not mean, however, that a very high PGPR concentration is necessarily better for high EE. In fact, the release of encapsulated compounds from the inner water droplets to the external aqueous phase can be due to coalescence of the inner water phase with the external water phase, or via diffusion through the oil phase, possibly via reverse micelles formed when there is an excess of lipophilic emulsifier in the oil phase.^7^, ^19^, ^20^

It is thus clear that some aspects still need to be elucidated in order to bring double emulsions towards food applications for soluble iron encapsulation: for instance, we should strive for minimizing PGPR concentration, since FAO regulations put a maximun on daily intake of 7.5 mg of PGPR per kilogram of body weight,^21^ while still ensuring the emulsion's physical stability. In addition, the effect of PGPR concentration on lipid oxidation in iron‐containing double emulsions has to be evaluated. Therefore, this research aimed to investigate the effect of PGPR concentration on the physicochemical stability of W_1_/O/W_2_ double emulsions targeted to ferrous sulphate encapsulation.

## MATERIAL AND METHODS

### Materials

Sunflower oil was purchased from a local supermarket (Wageningen, The Netherlands) and used without further purification. Ferrous sulphate heptahydrate was obtained from Merck Millipore (Darmstadt, Germany). Para‐anisidine of analytical grade, polyoxyethylenesorbitan monolaurate (Tween 20), and 2‐propanol were purchased from Sigma‐Aldrich (St Louis, MO, USA). PGPR was purchased from Quest International (Naarden, The Netherlands). d‐Glucose monohydrate was purchased from VWR Chemicals (Leuven, Belgium) and *n*‐hexane was purchased from Actu‐ALL Chemicals (Randmeer, The Netherlands). Acetic acid (glacial) 100% anhydrous was purchased from Merck Millipore (Darmstadt, Germany). Ultrapure water obtained from a Millipore Milli‐Q system (Darmstadt, Germany) was used throughout the study.

### Methods

#### *Preparation of W_1_/O/W_2_ double emulsions*

For making the primary water‐in‐oil (W_1_/O) emulsion, the aqueous phase contained 1 mol L^−1^ ferrous sulphate heptahydrate. The oil phase was prepared by mixing sunflower oil with different PGPR concentrations: 10, 25, 50, and 70 g kg^−1^ in the oil phase, at 200 rpm for 30 min at room temperature, followed by a 30‐min rest. Then, 25% *v*/*v* of the inner aqueous phase was drop‐wise dispersed into the oil phase using a rotor‐stator homogenizer (IKA® T18 Ultra Turrax, Staufen, Germany) at 11 000 rpm for 4 min.

The external aqueous phase was prepared with 2 mol L^−1^ glucose to balance the osmotic pressure difference. It was then added to 5 g kg^−1^ Tween 20 and stirred at 100 rpm for 1 h. To prepare a coarse W_1_/O/W_2_ double emulsion, 5% *v*/*v* of W_1_/O emulsion was drop‐wise dispersed into the external aqueous phase using a magnetic stirrer at 700 rpm for 15 min. The obtained coarse double emulsion was then passed through a premix membrane emulsification system three times.^22^ This emulsification system consists of a pressured vessel connected to a polymethyl methacrylate column (Wageningen University) with a nickel sieve placed between two rubber O‐rings at the bottom junction of the column. The nickel sieve had 500 μm thickness, 11.6 μm × 331 μm pore size and an effective area of 1.43 cm^2^ (Stork Veco B.V., Eerbeek, The Netherlands). The pressure vessel was connected to a nitrogen source and set to 400 kPa.

The freshly made W_1_/O/W_2_ double emulsions were kept in cylindrical plastic tubes at room temperature for further analysis. Samples to measure lipid oxidation were kept in the dark at room temperature.

#### *Droplet size distribution*

Droplet size distribution of the primary W_1_/O emulsion and W_1_/O/W_2_ double emulsion was determined by static light scattering (Mastersizer 2000, Malvern Instruments Ltd, Malvern, UK). In the case of the primary W_1_/O emulsion, sunflower oil was used as the continuous phase and the following conditions were applied: particle refractive index of 1.330, droplet absorbance of 0.01, dispersant refractive index of 1.465,^23^ obscuration between 5% and 20%. For the W_1_/O/W_2_ double emulsion, ultrapure water was used as a continuous phase and the following conditions were applied: particle refractive index of 1.465, droplet absorbance of 0.01, dispersant refractive index of 1.330, obscuration between 5% and 10%. The droplet size distribution was measured in fresh W_1_/O/W_2_ double emulsion and after 1 and 7 days of storage at room temperature. Before measuring, the samples were gently agitated to ensure homogeneity. Results were expressed as the Sauter mean diameter (*d*_32_) and span value (δ).

#### *Emulsion morphology*

Light microscopy (Carl Zeiss Axio Scope, Jena, Germany) images were taken in primary W_1_/O emulsions, and in fresh W_1_/O/W_2_ double emulsions, or after 1 and 7 days of storage at room temperature. The samples were gently agitated to ensure homogeneity. Then a drop of the sample was placed on a microscopic slide and covered with a slip. To visualize the primary emulsion the samples were diluted ten times with sunflower oil. Images were captured with an AxioCam MRc 5 camera at a magnification of ×400.

#### *Calculation of excess PGPR*

We calculated the theoretical excess fraction of PGPR (*E*_PGPR_) in a given volume of W_1_/O emulsion, considering a theoretical PGPR surface coverage (*Γ*_PGPR_) of 1.2 mg/m^2^ 24 (Eqn (1)):

(1) EPGPR%=mPGPRtot−3Vwaterr×ΓPGPR

where *m*_PGPR tot_ is the total mass of PGPR (in grams) in a given volume of emulsion, *V*_water_ is the volume of aqueous phase, and *r* is the water droplet radius (*d*_32_/2).

#### *Encapsulation efficiency*

The EE value was determined by direct conductivity measurements in the double emulsions (Hach HQ14d, Tiel, The Netherlands) according to Sahin *et al.* 22 The conductivity meter was placed into 20 mL vessels filled with the W_1_/O/W_2_ double emulsion and the emulsions were gently stirred. The conductivity was measured over time. The concentration of iron released in the external aqueous phase was determined using a calibration curve made with ferrous sulphate (0 to 13.7 × 10^−3^ mol L^−1^) in solutions of the same composition as the external aqueous phase (glucose and Tween 20).

The EE value was calculated based on the concentration that was released in the external water phase (*C*_w2_) relative to the maximum released concentration of iron (*C*_total_) (Eqn 2):

(2) $$\mathrm{EE}\;\left(\%\right)=\frac{\left(C_{\text{total}}-C_{w_{2}}\right)}{C_{\text{total}}}\;x\;100$$

where *C*_total_ was 12.9 × 10^−3^ mol L^−1^, corresponding to a theoretical situation where all the iron would have been released in the external aqueous phase.

#### *Lipid oxidation*

Lipid oxidation was determined by measuring the amount of conjugated diene (CD) hydroperoxides^25^ and the aldehyde content (mainly alkene‐2‐als) through the para‐anisidine value (pAV).^26^ The pAV protocol was slightly modified according to Berghout *et al.* 27 and Cengiz *et al.* 28

For the determination of CD hydroperoxides, 50 μL of W_1_/O/W_2_ double emulsion were mixed with 950 μL 2‐propanol. This first sample was further diluted ten‐fold with 2‐propanol. The obtained sample was centrifuged at 1200×*g* for 4 min. The absorbance spectrum of the supernatant was recorded between 200 and 310 nm with a UV‐visible spectrophotometer (DU720, Beckman Coulter, Inc., Indianapolis, IN, USA), using a blank consisting of 2‐propanol and water in the same ratio as in the measured sample. Measurements were done on fresh emulsions, after 4 h, 1 day and 7 days storage at room temperature. The concentration of the CD hydroperoxides was calculated using the measured absorbance at 233 nm and their molar extinction coefficient at 233 nm (27 000 M^−1^ cm^−1^), and expressed as mmol kg^−1^ of oil.

The determination of the pAV started with weighing 1 g of W_1_/O/W_2_ double emulsion. Then the emulsion was mixed with 2.5 mL hexane/isopropanol (3:1, *v*/*v*). The upper hexane phase was centrifuged at 1200×*g* for 4 min and the supernatant was collected to measure the absorbance at 350 nm (Ab), using pure hexane as the blank. Then, 0.5 mL of the upper hexane phase was mixed with 0.1 mL of 2.5 g L^−1^ para‐anisidine in acetic acid solution. The blank was hexane mixed with the same para‐anisidine solution, in similar proportions. After 10 min the absorbance (As) of the samples was measured at 350 nm. The pAV was calculated as Eqn (3):

(3) $$\mathrm{pAV}=\frac{\left(1.2\mathrm{As}-\mathrm{Ab}\right)}{m}$$

where *m* is the mass of oil per millilitre of hexane phase.

Double emulsions without iron were prepared as a control. Besides, a simple oil‐in‐water (O/W) emulsion (37.5 mL L^−1^ sunflower oil, 5 g kg^−1^ Tween 20 and 2 mol L^−1^ glucose in the aqueous phase) was prepared with 12.9 × 10^−3^ mol L^−1^ iron in the aqueous phase (i.e. same as *C*_total_ used for EE measurements). Iron was dispersed at the aqueous phase under magnetic stirring at 100 rpm. The aqueous phase was prepared as described earlier. The oil was drop‐wise dispersed into the aqueous phase under magnetic stirring at 700 rpm for 15 min. The obtained coarse emulsion was then passed through a premix membrane emulsification system three times using the same parameters as described in earlier. This mixture corresponds to a situation in the W_1_/O/W_2_ double emulsion where all the water and iron would have been released from the inner water droplets to the external aqueous phase.

#### *Experimental design and statistical analysis*

For each PGPR concentration, three double emulsion samples were prepared independently. To determine droplet size distributions, each sample was measured twice with an average of four readings. Microscopy images and EE were analysed in duplicate per sample. Lipid oxidation measurements were taken in triplicate per each sample. Data are presented as a mean and standard deviation. Statistical analysis was done using the IBM SPSS software v. 23 (Statistical Package for the Social Sciences, SPSS Inc., Chicago, IL, USA). The normality of the data was tested with Kolmogorov–Smirnov test. Means from samples prepared with different PGPR concentrations within the same storage time were compared by one‐way analysis of variance (ANOVA) with Tukey's *post hoc* test, with a significance level of *P* < 0.05.

## RESULTS AND DISCUSSION

The physicochemical stability of W_1_/O/W_2_ double emulsions prepared with different PGPR concentrations was monitored over storage time at room temperature. The physical stability was assessed considering droplet size distribution, microscopy images, and EE. The formation of primary and secondary lipid oxidation compounds was used to analyse chemical stability.

### Physical properties of W_1_/O emulsions

**Figure 1:**
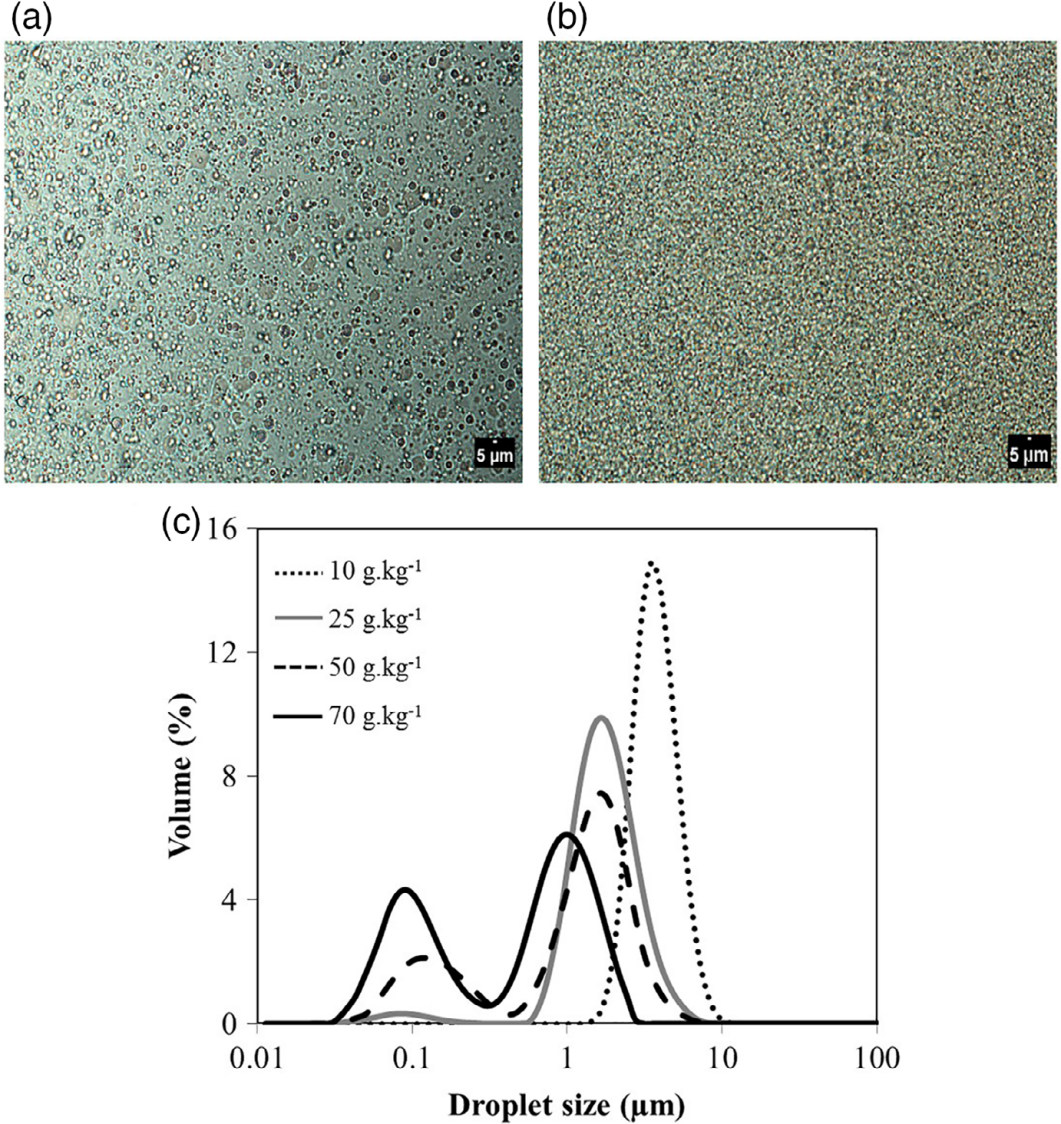
Microscopy images of fresh primary water‐in‐oil (W_1_/O) emulsions prepared with 10 g kg^−1^ (a) and 70 g kg^−1^ (b) polyglycerol polyricinoleate (PGPR). Droplet size distribution of W_1_/O emulsions prepared with different PGPR concentrations in the oil phase (c): 10, 25, 50 and 70 g kg^−1^.

Figure 1(a) and (b) shows optical microscopy images of W_1_/O emulsions prepared with the lowest and the highest PGPR concentrations tested. It is clear that W_1_/O emulsions prepared with 70 g kg^−1^ PGPR showed smaller droplets compared to W_1_/O with 10 g kg^−1^ PGPR. Figure 1(c) shows the droplet size distribution of W_1_/O emulsions prepared with different PGPR concentrations. A bimodal distribution was seen for W_1_/O emulsions with higher PGPR concentrations, with a first peak between 0.1 and 0.14 μm and a second peak between 1 and 1.5 μm. The polydispersity also increased when PGPR concentration was increased (Table 1). Emulsions prepared with 50 and 70 g kg^−1^ PGPR had the smallest *d*_32_ and the highest span values compared to lower PGPR‐concentrations.

**Table 1:** Sauter mean diameter (*d*_32_), span values and excess of polyglycerol polyricinoleate (PGPR) (%) in primary water‐in‐oil (W_1_/O) emulsions prepared with various PGPR concentrations

PGPR concentration (g kg^−1^)	*d*_32_ (μm)	Span	Excess of PGPR (%)
10	3.33 ± 0.29a	0.84 ± 0.04	92.8
25	1.45 ± 0.43b	1.30 ± 0.59	93.4
50	0.76 ± 0.61bc	5.74 ± 6.62	93.7
70	0.21 ± 0.17c	2.28 ± 0.37	83.7

Results of *d*_32_ and span are expressed as mean ± standard deviation (*n* = 3). Lowercase letters indicate significant (*P* < 0.05) differences in *d*_32_ between W_1_/O emulsions prepared with different PGPR concentrations.

Márquez *et al.* 29 also described a decrease in *d*_32_ and an increase in polydispersity in W/O emulsions prepared with increasing PGPR concentrations. Scherze *et al.* 30 also observed that increasing PGPR concentration (25 to 40 g kg^−1^ in the oil phase) resulted in decreasing the droplet size of W/O emulsions. To make physically stable double emulsions, it is preferable to have W_1_/O emulsions with small droplet size, to avoid rapid droplet sedimentation. In fact, a slower sedimentation rate can prevent the water droplets coming into close contact with the sedimentation layer, which reduces coalescence rates.^31^

The peak around 0.1 μm could correspond to reverse micelles (Fig. 1(c)) when there is an excess of PGPR in the oil phase. Ushikubo and Cunha^31^ also found a bimodal droplet size distribution with the first peak mode around 0.1 and 0.2 μm, in W/O emulsions prepared with PGPR and soybean oil. The authors argue that the first peak could represent the excess of emulsifier that is free or present as small aggregates in the continuous phase. The formation of reverse micelles in the oil phase has been described by other authors.^4^, ^32^ We calculated that PGPR was most likely present in large excess (83.7% to 93.7%) for all tested concentrations (Table 1). Nollet *et al.* 33 determined the critical micellar concentration of PGPR in sunflower oil, which they found to be 10 g kg^−1^. This would imply that in our case, emulsions made with PGPR concentration equal to or above 25 g kg^−1^ would contain excess PGPR as reverse micelles in the oil phase. Moreover, the ability of free PGPR to aggregate and form reverse micelles has been described by the formation of spontaneous water droplets in oil phase even at low PGPR concentration (5 g kg^−1^) without homogenization.^34^

### Physical properties and stability of W_1_/O/W_2_ double emulsions

#### *Droplet size distribution*

**Figure 2:**
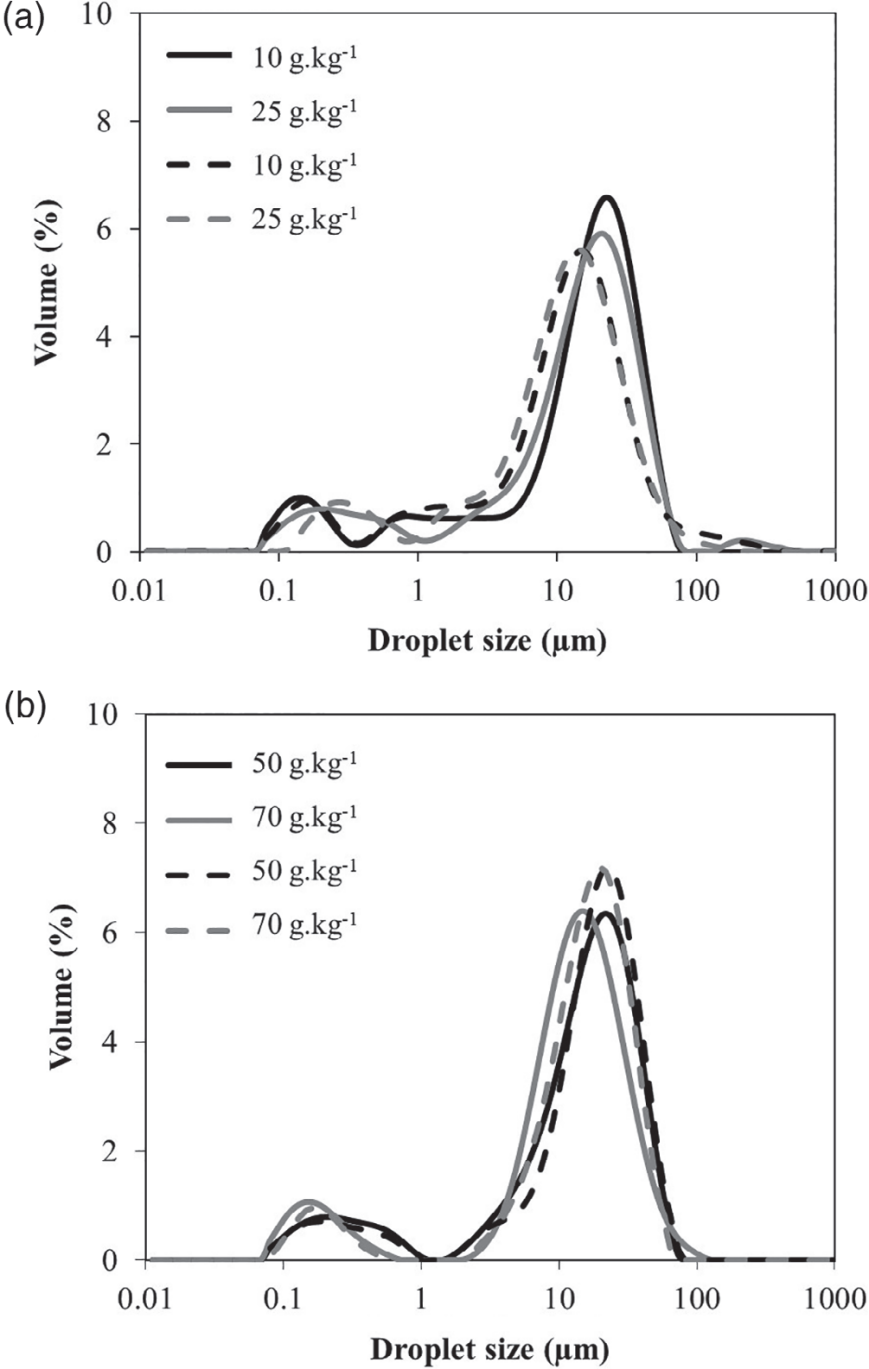
Droplet size distribution of fresh water‐in‐oil‐in‐water (W_1_/O/W_2_) double emulsions (solid line), and after 7 days (dashed line) of storage, prepared with different polyglycerol polyricinoleate (PGPR) concentrations in the oil phase: (a) 10 and 25 g kg^−1^, (b) 50 and 70 g kg^−1^.

**Figure A1:**
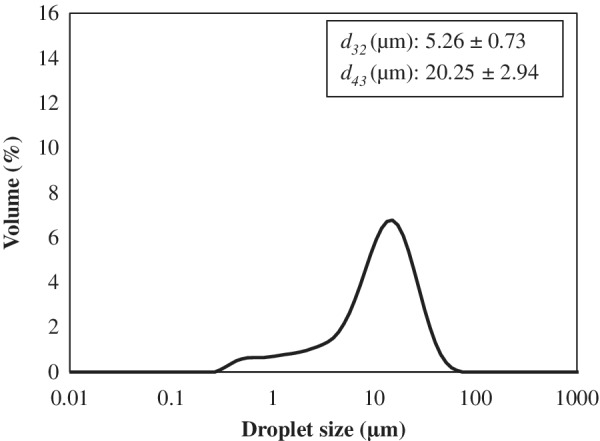
Droplet size distribution of fresh oil‐in‐water (O/W) emulsions with no polyglycerol polyricinoleate (PGPR), prepared with homogenization conditions similar to the second emulsification step for double emulsions (representative curve from measurements of three independent emulsions).

The droplet size distributions of W_1_/O/W_2_ double emulsions prepared with different PGPR concentrations were monitored over time at room temperature. All fresh double emulsions had a bimodal droplet size distribution (Fig. 2(a,b)) and a main peak around 17 μm with only slight changes depending on the PGPR concentration. The peak seen between 0.1 and 1 μm can correspond to the scattering of inner water droplets. In a comparable way, Kaimainen *et al.* 35 described a first peak at 0.31 μm and a second at 6.6 μm in the droplet size distribution of double emulsions. It was assumed that the first peak corresponded to the primary emulsion droplets (3 *v*/*w*% W_1_/O in the double emulsions). Dickinson *et al.* 36 argued that the complex geometry of double emulsions complicates the interpretation results of light scattering analysis because the results are obtained based on the assumption that the inner water droplets do not significantly change the refractive index of the oil droplets**.** Therefore, those measurements are useful to obtain an estimation about the size and size distribution of the droplets but should be interpreted with caution. We checked the particle size distribution of O/W emulsions prepared in the same conditions as the double emulsions, except that the dispersed phase was now only oil, instead of a W/O emulsion. The particle size distribution and corresponding *d*_32_ and *d*_43_ values are presented in the Appendix (Fig. A1). As expected, the particle size distribution of these O/W emulsions presented the same main peak as the W/O/W emulsions, centred around 20 μm, which is thus characteristic of the oil droplets. Although some smaller sizes were also detected, the small submicron droplet peak that was seen for the double emulsions was not present for the O/W emulsions, confirming that this small peak indeed corresponds to the inner water droplets.

There was a slight left‐shift of the peak of double emulsions prepared with low PGPR concentrations after 7 days of storage at room temperature, which indicates a decrease in droplet diameter at these PGPR concentrations **(**Fig. 2(a)). This effect could possibly be due to the loss of the inner water droplets. The latter represents 25% of the volume of the oil droplets, meaning that, if this entire volume would be released to the outer aqueous phase, it would result in a 9%‐decrease in the oil droplet diameter.

#### *Morphology*

**Figure 3:**
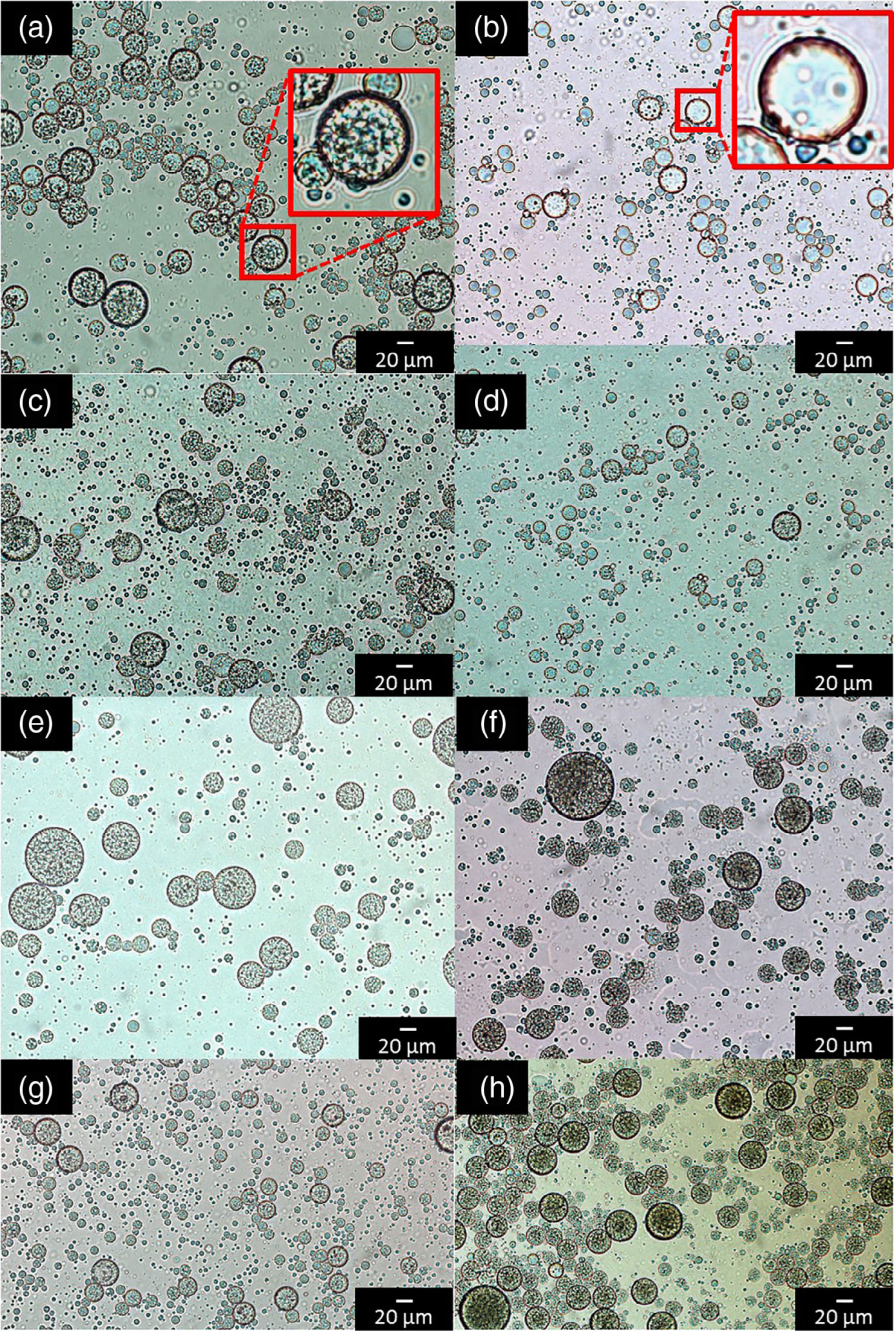
Microscopy images of water‐in‐oil‐in‐water (W_1_/O/W_2_) double emulsions freshly prepared (left) and after 7 days of storage (right) containing 10 g kg^−1^ PGPR (a, b), 25 g kg^−1^ PGPR (c, d), 50 g kg^−1^ PGPR (e, f) and 70 g kg^−1^ PGPR (g, h). On panels (a) and (b), the red squares show a magnification of typical droplets.

The morphology of double emulsions was studied by light microscopy, on fresh emulsions and emulsions after 7 days of storage (Fig. 3). The inner water droplets could be clearly observed in the fresh emulsions (Fig. 3(a,c,e,g)). However, after 7 days of storage, those inner droplets were not clearly visible any more in the double emulsion with 10 g kg^−1^ PGPR (Fig. 3(b)). This suggests that the inner water droplets were not stable in this sample and that they were largely expelled to the external aqueous phase. A similar effect was observed, yet to a lower extent, in the double emulsions with 25 g kg^−1^ PGPR (Fig. 3(d)). These results could explain the left‐shift of the main peak observed in the droplet size distribution of double emulsions prepared with low PGPR concentrations during storage (Fig. 2(a)). Conversely, the morphology of double emulsions with higher PGPR concentrations looked stable over storage (Fig. 3(f,h)). These results suggest that a certain minimum PGPR concentration is required to keep the inner water droplets stable along storage, even when no large osmotic pressure gradient is present.

#### *Encapsulation efficiency (EE)*

**Figure 4:**
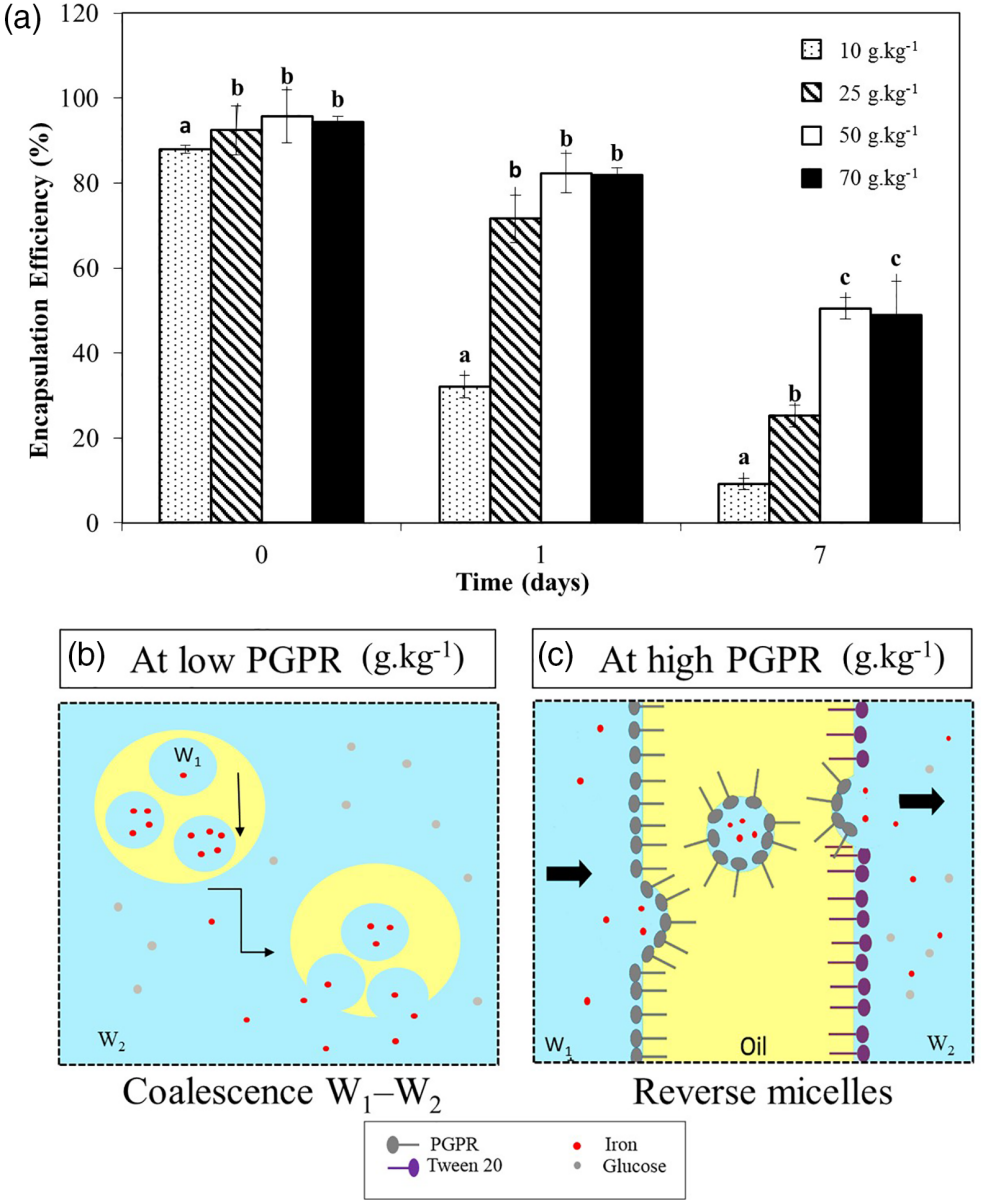
Encapsulation efficiency (%) of water‐in‐oil‐in‐water (W_1_/O/W_2_) double emulsions prepared with different polyglycerol polyricinoleate (PGPR) concentrations in the oil phase, over storage time. Results are expressed as mean (*n* = 3) and standard deviation as error bars. Different letters means significant difference at *P* < 0.05, within the same storage time (a). Proposed mechanisms of iron release in double emulsions stabilized with different PGPR concentrations. Iron release in double emulsions with low PGPR concentration (10–25 g kg^−1^), mainly by the coalescence of the inner aqueous phase (W_1_) with the external aqueous phase (W_2_) (b). Iron release in double emulsions with high PGPR concentration (50–70 g kg^−1^), mainly by the presence of reverse micelles in the oil phase (c).

The EE value was determined in fresh double emulsions and after storage (Fig. 4(a)). All fresh double emulsions presented an EE between 88% and 96%, depending on the PGPR concentration. After 1 day of storage, double emulsions prepared with 10 g kg^−1^ PGPR showed a decrease in the EE to 32%, which further decreased to less than 10% after 7 days of storage. Double emulsions prepared with high PGPR concentrations (50 and 70 g kg^−1^) retained most of the iron encapsulated after 1 day. However, after 7 days of storage, the EE decreased to ∼50% in these double emulsions. Therefore, it seems that low PGPR concentrations lead to rapid release of iron, but that beyond a certain PGPR concentration (here, 50 g kg^−1^) no further improvement can be achieved from an EE point of view. The pronounced release of iron in double emulsions prepared with low PGPR concentration after 7 days may be explained by sedimentation of larger water droplets within the oil droplets. Such a sedimentation would bring the water droplets closer to each other, and to the oil droplet surface, facilitating the release of water and iron to the external aqueous phase (Fig. 4(b)), which could explain the change in droplet morphology seen in Fig. 3(a–d). Conversely, for high PGPR concentrations, we hypothesize that iron was predominantly released by reverse micelles, giving lower rates of iron release (Fig. 4(c)). Choi *et al.* 7 described similar conclusions for iron transport in double emulsions prepared with 80 g kg^−1^ PGPR. The authors assumed that most of the water droplets remained within the oil droplets since there was no significant change in *d*_43_ or in morphology over 7 days of storage. Therefore, most likely iron was transported by reverse micelles. In our work, we excluded the possibility of iron release due to insufficient coverage of the W_1_/O interface since for all PGPR concentrations there was largely enough emulsifier to cover all the water droplets.

To sum up, PGPR concentration can affect EE in double emulsions during storage via two effects: first, a minimal PGPR concentration is needed to ensure small enough water droplets that do not readily coalesce with the external aqueous phase. However, a too high a PGPR concentration is not recommended, as a large amount of reverse micelles in the oil phase can participate in releasing iron to the external aqueous phase.

### Chemical stability of W_1_/O/W_2_ double emulsions: lipid oxidation

The formation of primary lipid oxidation compounds was assessed by measuring CD hydroperoxides, and that of secondary lipid oxidation compounds by determining total aldehydes (pAV).

**Figure 5:**
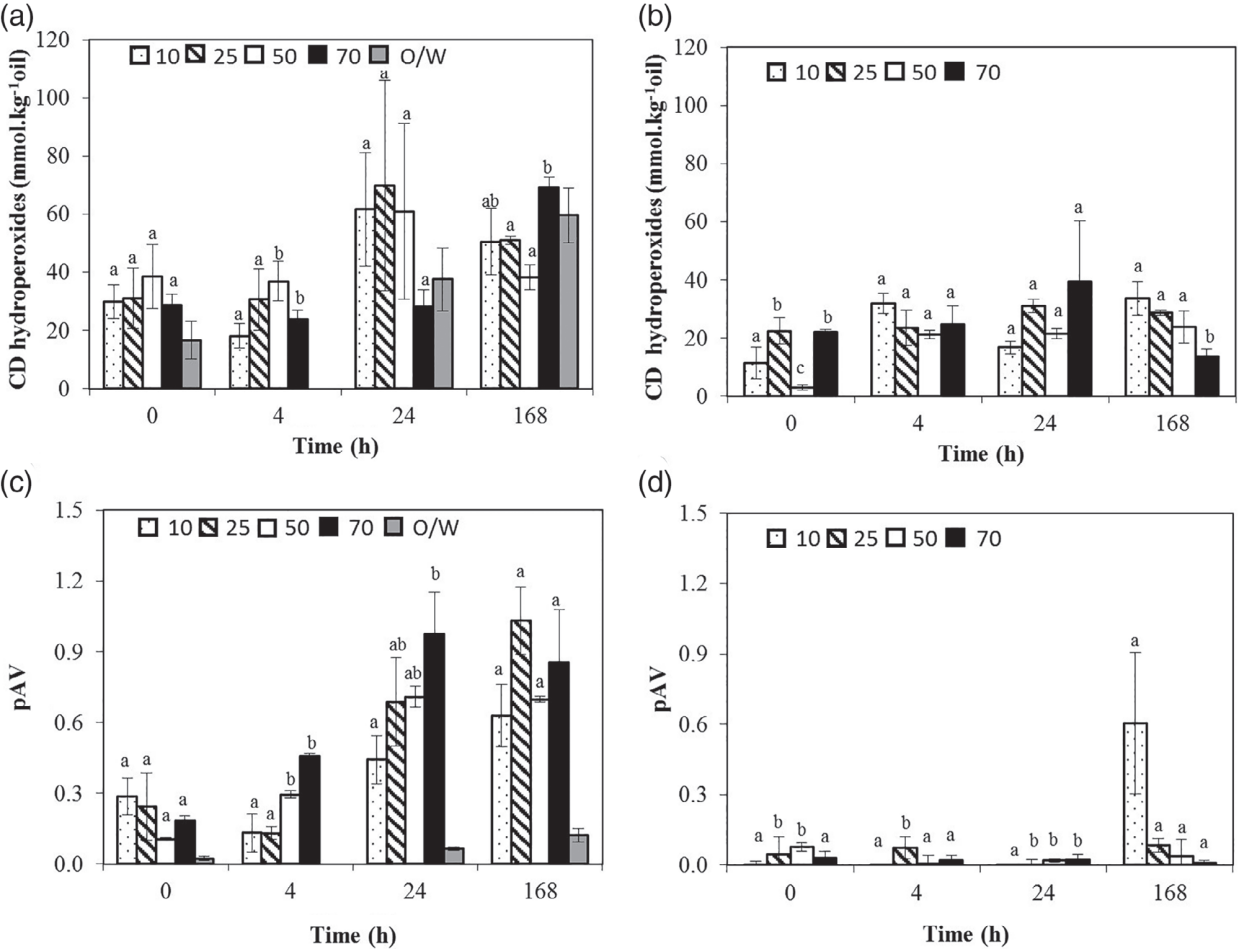
Lipid oxidation in double emulsions prepared with different polyglycerol polyricinoleate (PGPR) concentrations in the oil phase (g kg^−1^), and in a control oil‐in‐water (O/W) emulsion over 168 h storage at room temperature. Conjugated diene (CD) hydroperoxides (mmol kg^−1^ oil) in double emulsions and O/W emulsion with iron (a) and without iron (b); para‐anisidine value (pAV) in double emulsions and O/W emulsion with iron (c) and without iron (d). Results are expressed as mean (*n* = 3) and standard deviation as error bars. Different letters mean significant difference at *P* < 0.05, within the same storage time.

Lipid oxidation was first monitored in double emulsions without iron. In those emulsions, the initial CD hydroperoxide concentration was low, ranging from about 3 to 23 mmol kg^−1^ (Fig. 5(b)). Moreover, the pAV of these double emulsions (Fig. 5(d)) was very low, independently of the PGPR concentration. The CD hydroperoxide concentration in fresh sunflower oil was previously reported as 17.4 mmol equivalents CD hydroperoxides per kilogram of oil.^37^ The low pAV found was expected in the absence of iron, since iron can decompose CD hydroperoxides, which is the starting point for the formation of secondary lipid oxidation products.^38^ Overall, during storage, lipid oxidation in double emulsions without iron did not increase substantially PGPR concentrations at all.

The presence of iron in the water droplets increased the formation of CD hydroperoxides (29–39 mmol kg^−1^) in fresh double emulsions compared to double emulsions without iron, independently of the PGPR concentration (Fig. 5(a)). The high CD hydroperoxide concentrations in fresh double emulsions with iron could indicate that lipid oxidation has already happened during the emulsification process. The incorporation of oxygen during the emulsification process has been reported to induce lipid oxidation.^39^, ^40^ Despite the presence of natural antioxidants in the oil, we did not observe a lag phase of oxidation in double emulsions, both without and with iron.

The formation of CD hydroperoxides was monitored over time in double emulsions with iron. After 4 h of storage, no substantial formation of CD hydroperoxides in double emulsions with iron could be detected, but after 24 h of storage CD hydroperoxide concentration increased significantly in double emulsions with 10 to 50 g kg^−1^ PGPR. Interestingly, at the highest PGPR concentration (70 g kg^−1^) the CD hydroperoxides only increased considerably after 168 h of storage.

Nevertheless, there was no difference in the pAV in the fresh emulsions prepared with various PGPR concentrations (Fig. 5(c)). However, there was a more noticeable increase in pAV after 4 h of storage in double emulsions with higher PGPR concentrations (50 and 70 g kg^−1^) compared to double emulsions with lower PGPR concentrations, which continued to increase over time. At low PGPR concentrations, there was a significant increase only after 24 h of storage. For all double emulsions, the pAV seemed to level off after 24 h of storage.

The increase in lipid oxidation observed in our study was attributed to the presence of iron in the double emulsions, since it was substantially promoted compared to double emulsions with no iron. It is well known that the redox cycling of iron is a relevant mechanism to accelerate lipid oxidation. Ferrous iron (Fe^2+^) in emulsion systems can accelerate the decomposition of pre‐existing hydroperoxides, forming ferric iron (Fe^3+^) and alkoxyl radicals. Furthermore, Fe^3+^ can further react with hydroperoxides to form Fe^2+^ and peroxyl radicals.^41^, ^42^ These radicals can react with unsaturated lipids within the droplet or at the interface, leading to the formation of new lipid radicals, thereby propagating lipid oxidation. Kristinova *et al.* 43 described that the addition of Fe^2+^ to O/W emulsions rapidly decreased the oxygen concentration compared to the addition of Fe^3+^, due to the fast rate of oxidation of Fe^2+^ to Fe^3+^ by pre‐existing lipid hydroperoxides in the system. This can explain the fast decomposition of CD hydroperoxides seen for double emulsions with iron, followed by a level‐off.

In addition, the structure of the emulsion can affect the pro‐oxidant effect of iron. First, the emulsifier charge can affect iron location within the system. For instance, Mancuso *et al.* 44 have shown that anionic surfactants attract iron ions to the droplet surface, increasing iron interaction with hydroperoxides. Second, surfactant micelles in the continuous phase may segregate iron and decrease lipid oxidation.^45^ In our study, we assume that at low PGPR concentration most of the iron was released to the external aqueous phase during storage, which may have minimized contact with hydroperoxides present in the oil. Conversely, at high PGPR concentrations most of the iron remained encapsulated over time (Fig. 4(a)), which promoted contact between iron and hydroperoxides. Third, the droplet size is also of importance: a larger interfacial area (smaller droplets) is often associated with a higher lipid oxidation rate due to an increased contact area between the oil and aqueous phase pro‐oxidants. In our double emulsions, increasing PGPR concentration led to a substantial decrease of the inner water droplet size (Table 1), which resulted in a larger interfacial area. However, the effect of interfacial area and droplet size on oxidative stability is still contradictory in the literature and some studies have reported better oxidative stability in O/W emulsions with smaller droplets.^39^, ^46^ Therefore, there is evidence that the droplet size and interfacial area can affect lipid oxidation, but the mechanism of the reaction will depend on the emulsion composition.

To test the potential effect of iron release in the external aqueous phase on lipid oxidation, we prepared simple O/W emulsions with iron present in the aqueous phase, to mimic a situation where all the inner water droplets would have been released to the external aqueous phase. We noticed that there was an increase in the formation of CD hydroperoxides over time (Fig. 5(a)), as much as for double emulsions with iron. Thus, the ability of iron to generate primary lipid oxidation products was found not only when present in the inner water droplets of the double emulsions but also when present in the external aqueous phase. However, the pAV in O/W emulsions was lower than for all double emulsions containing iron. Therefore, we concluded that iron is less efficient at decomposing hydroperoxides into secondary oxidation products when diluted in the external aqueous phase than when concentrated in the inner water droplets. A previous study by Choi *et al.* 7 also investigated the effect of iron location on lipid oxidation in a double emulsion‐based system: the authors measured lipid oxidation in a simple fish O/W emulsion, to which a double W_1_/O/W_2_ emulsion containing iron, made with a less oxidizable oil (corn oil) was added. Surprisingly, they found that when iron was encapsulated in the inner droplets of the W_1_/O/W_2_ emulsion, i.e. separated from the O/W fish oil droplets, the latter oxidized more than when iron was present in the external water phase, i.e. in direct contact with the fish oil droplets. Although no obvious explanation for this finding could be proposed, the authors hypothesized that corn oil itself could have oxidized because of the presence of iron in the inner water droplets, which could have catalysed the subsequent oxidation of the fish oil, although the latter was not in direct contact with iron.

Finally, it has also been described that surfactant micelles can remove hydroperoxides from the interface and limit lipid oxidation.^43^ Chen *et al.* 47 described a reduction in the lag phase formation of lipid hydroperoxides in the presence of reverse micelles formed with phospholipids in bulk oil, which indicates the pro‐oxidant activity of reverse micelles. Conversely, Yi *et al.* 48 described that without iron, the oxidative stability of W/O emulsions was improved at higher PGPR concentrations (3–10 g kg^−1^), due to reverse micelles formed with non‐adsorbed PGPR that removed hydroperoxides from the droplet surface. When iron (Fe^2+^) was added to the W/O emulsions the hydroperoxides decomposition went faster at lower PGPR concentrations, which supports the hypothesis of the partitioning of hydroperoxides into reverse micelles.

## CONCLUSIONS

The present study investigated the physical and chemical stability of double emulsions containing iron in the inner water droplets. Regarding the physical stability, even a high PGPR concentration was not sufficient to fully prevent the release of iron to the external aqueous phase over storage. Yet increasing PGPR concentration did help in some respect, but only up to a certain concentration, above which no further increase in EE could be obtained. We hypothesized that the transport of iron was mostly due to the coalescence of the inner water droplets with the external aqueous phase at low PGPR concentration, and mostly due to transport via reverse micelles at high PGPR concentrations. The PGPR concentration also had an effect on lipid oxidation. The formation of CD hydroperoxides increased over time in all double emulsions, and pAV increased more substantially in double emulsions with 70 g kg^−1^ PGPR concentration. Double emulsions with high PGPR concentration had smaller water droplets, thus a larger interface area that probably promoted lipid oxidation. We conclude that from a physical stability perspective, a high PGPR concentration was enough to keep most of the iron encapsulated before 7 days of storage, after which the EE decreased. From a chemical stability perspective, all double emulsions were unstable. A higher PGPR concentration increased the formation of secondary lipid oxidation products. Therefore, we assumed that the physicochemical changes observed in these double emulsions certainly hamper their suitability as iron encapsulation systems. An optimal PGPR concentration needs to be combined with strategies to reduce iron lipid oxidation and increase EE. This could next be a basis to tailor a physicochemical stable W_1_/O/W_2_ double emulsion for iron encapsulation.

## DECLARATION OF INTEREST STATEMENT

The authors declare no competing financial interest.
